# Effect of Tumor Necrosis Factor Inhibitor Therapy on Osteoclasts Precursors in Ankylosing Spondylitis

**DOI:** 10.1371/journal.pone.0144655

**Published:** 2015-12-16

**Authors:** Inês P. Perpétuo, Rita Raposeiro, Joana Caetano-Lopes, Elsa Vieira-Sousa, Raquel Campanilho-Marques, Cristina Ponte, Helena Canhão, Mari Ainola, João E. Fonseca

**Affiliations:** 1 Rheumatology Research Unit, Instituto de Medicina Molecular, Faculdade de Medicina da Universidade de Lisboa, Lisbon Academic Medical Centre, Lisboa, Portugal; 2 Rheumatology and bone metabolic diseases department, Hospital de Santa Maria, Centro Hospitalar Lisboa Norte, EPE, Lisbon Academic Medical Centre, Lisboa, Portugal; 3 Musculoskeletal Diseases and Inflammation Research Group, Biomedicum Helsinki, Faculty of Medicine, Institute of Clinical Medicine, University of Helsinki, Helsinki, Finland; INSERM - university Paris 7, FRANCE

## Abstract

**Introduction:**

Ankylosing Spondylitis (AS) is characterized by excessive local bone formation and concomitant systemic bone loss. Tumor necrosis factor (TNF) plays a central role in the inflammation of axial skeleton and enthesis of AS patients. Despite reduction of inflammation and systemic bone loss, AS patients treated with TNF inhibitors (TNFi) have ongoing local bone formation. The aim of this study was to assess the effect of TNFi in the differentiation and activity of osteoclasts (OC) in AS patients.

**Methods:**

13 AS patients treated with TNFi were analyzed at baseline and after a minimum follow-up period of 6 months. 25 healthy donors were recruited as controls. Blood samples were collected to assess receptor activator of nuclear factor kappa-B ligand (RANKL) surface expression on circulating leukocytes and frequency and phenotype of monocyte subpopulations. Quantification of serum levels of bone turnover markers and cytokines, *in vitro* OC differentiation assay and qRT-PCR for OC specific genes were performed.

**Results:**

RANKL^+^ circulating lymphocytes (B and T cells) and IL-17A, IL-23 and TGF-β levels were decreased after TNFi treatment. We found no differences in the frequency of the different monocyte subpopulations, however, we found decreased expression of CCR2 and increased expression of CD62L after TNFi treatment. OC number was reduced in patients at baseline when compared to controls. OC specific gene expression was reduced in circulating OC precursors after TNFi treatment. However, when cultured in OC differentiating conditions, OC precursors from AS TNFi-treated patients showed increased activity as compared to baseline.

**Conclusion:**

In AS patients, TNFi treatment reduces systemic pro osteoclastogenic stimuli. However, OC precursors from AS patients exposed to TNFi therapy have increased *in vitro* activity in response to osteoclastogenic stimuli.

## Introduction

Ankylosing spondylitis (AS) is a systemic, chronic, immune-mediated inflammatory disease that affects the musculoskeletal system. The axial skeleton and enthesis are predominantly involved in this disease and tumor necrosis factor (TNF) seems to play a central role [1]. AS is characterized by local excessive bone formation, but it is also associated with systemic bone loss, which is a common complication even in the early stages of the disease [2].

The immune and skeletal systems have several common regulatory factors and immune system cells have a profound influence on bone metabolism, particularly in chronic inflammatory diseases. Receptor activator of nuclear factor κB ligand (RANKL) is present on osteoblasts surface, but is also expressed by activated immune cells, both in its membrane form and as a soluble molecule [3]. Cytokines such as TNF, interleukin (IL)-1β, IL-6 and IL-17 are secreted by activated immune cells and act synergistically with the RANK-RANKL system [4,5], further enhancing osteoclast (OC) differentiation from its circulatory precursors (monocytes) and contributing to bone resorption [1,3]. Monocytes are phenotypically and functionally heterogeneous and have a critical regulatory role in inflammation and innate immune responses [6]. Three sub-populations of monocytes have been described in humans, based on their expression of CD14 and CD16 surface markers. The classical subset, CD14^bright^CD16^-^ accounts for 85% of monocytes, includes phagocytic cells and OC precursors; the non-classical subset CD14^dim^CD16^+^ accounts for 10% of monocytes and is involved in cytokine production and T-cell activation. The intermediate, the most recently described subset, accounts for only 5% of monocytes and is CD14^bright^CD16^+^. This latter subset is considered to be the antigen presenting subset and is responsible for reactive oxygen species production [6]. Monocytes are key players in immune-mediated inflammatory diseases and their excessive and sustained activity is a hallmark of AS [7].

Serum levels of TNF, IL-6 and IL-17 are increased in AS patients, which may contribute to the well documented secondary osteoporosis that occur in these patients [1,8]. TNFi are very effective in the mitigation of inflammation in AS patients and induce a reduction in CTX-I levels, which may reflect a decrease in OC activity [8]. The aim of this study was to assess the effect of TNFi in the differentiation and activity of OC precursors in AS patients.

## Patients and Methods

### Patients

The local ethics committee (Hospital de Santa Maria) approved this study and all participants signed an informed consent. Patients were managed in accordance with the standard practice and the study was conducted in accordance with the Declaration of Helsinki as amended in Brazil (2013). Patients with AS fulfilling the New York modified criteria 1984 [9] were recruited from the Rheumatology and Bone Metabolic Disease Department, Lisbon Academic Medical Centre, Portugal. All patients were included before starting the first TNFi and were followed-up during a minimum period of 6 months after initiating therapy. Other inclusion criteria at baseline were: active disease, defined as AS disease activity score (ASDAS-ESR)>1.3 [10] and documented axial involvement by X-ray or magnetic resonance imaging (MRI). Patients previously exposed to biological TNFi were excluded. Information regarding patients’ demographics, duration of symptoms, peripheral involvement, syndesmophyte formation, HLA-B27 positivity, erythrocyte sedimentation rate (ESR) and C-reactive protein (CRP) was collected. ASDAS was evaluated, as well as the Bath Ankylosing Spondylitis Disease Activity Index (BASDAI [11]) and the Bath Ankylosing Spondylitis Functional Index (BASFI [12]). Heparinized blood and serum were collected from each participant at the starting date of TNFi and another collection was made after a minimum period of 6 months of follow-up. Blood was used for flow cytometry and peripheral blood mononuclear cell (PBMC) isolation. Donors matched for age and gender were used as controls. Samples were stored and managed by the Biobanco-IMM, Lisbon Academic Medical Center, Lisbon, Portugal. The local ethics committee approved this study and all participants signed an informed consent.

### Flow cytometry

Identification of B and T cells and granulocytes in peripheral blood and immunophenotyping of monocytes in the PBMC samples were performed using matched combinations of anti-human murine mAbs. For peripheral blood staining anti-CD19 PerCP-Cy5.5 (eBioscience), anti-CD3 PerCP (BD Biosciences), anti-CD66b FITC (Immunotools) and anti-RANKL PE (Santa Cruz Biotechnology) were used. Monocyte subpopulations were identified with anti-CD14 FITC (BD Biosciences) or PerCPCy5.5 (Immunotools) and anti-CD16 APC (Immunotools) and stained with combinations of anti-CD11b PE-Cy7, CD105 PE, CD62L PE-Cy7, CD51/CD61 FITC (eBioscience), CCR2 PE (R&D Systems), HLA-DR PerCP (BD Biosciences) and RANK PE (Santa Cruz Biotechnology). Cell death was assessed by staining with Annexin V Apoptosis Detection Kit APC (eBioscience). Acquisition was performed using a FACSCalibur (BD Biosciences).

Heparinized whole blood was used for staining. Erythrocytes were lysed with red blood cell lysis buffer and cells were incubated with IgG block solution 300ng/mL (ChromPure Mouse IgG whole molecule, Jackson ImmunoResearch Laboratories) before staining. Absolute cell counts were calculated from differential leukocyte count determined for all participants. PBMCs were isolated by density gradient centrifugation with Histopaque^®^-1077 (Sigma-Aldrich). Monocyte subpopulations were identified as described before based on their CD14 and CD16 surface expression [6]. Data was analyzed using FlowJo software (TreeStar, Stanford University).

### Cytokine detection in the serum

IL-1β, IL-6, IL-12(p70), IL-17A, IL-23, monocyte chemotactic protein-1 (MCP-1), transforming growth factor-beta (TGF-β) and TNF levels were measured in the serum by FlowCytomix custom assay kits (Bender MedSystems) according to the manufacturer instructions. Samples were acquired with a FACS Calibur flow cytometer (BD Biosciences). Raw data of the flow cytometry bead assay were analyzed by FlowCytomix Pro 3.0 software (Bender MedSystems). Carboxy-terminal type I collagen crosslinks (CTX-I), human type I procollagen amino-terminal-propeptide (P1NP, Sunred Biological technology), osteoprotegerin (OPG), sclerostin (SOST), dickkopf-related protein (DKK)-1 and soluble RANKL (ampli-sRANKL, Biomedica Grouppe) were quantified by enzyme-linked immunosorbent assay (ELISA) in serum samples according to the manufacturer’s instructions.

### PBMC isolation and cell culture

PBMCs were isolated by density gradient centrifugation and plated in 96-well culture plates at a density of 7.0x10^5^ cells/well and in 24-well culture plates at a density of 1.5x10^6^ cells/well in Dulbecco's Modified Eagle Medium (DMEM; Invitrogen) supplemented with 5000 U Penicilin/Streptomicin (Invitrogen), 2 mM L-Glutamine (Invitrogen) and 10% Fetal Bovine Serum (FBS; Invitrogen) and incubated in a humidified atmosphere at 37°C, 5% CO_2_. PBMCs were left overnight for OC precursors (OCPs) to adhere on bone slices. On the following day (day 1 of culture) medium was changed to DMEM supplemented with M-CSF 25 ng/mL (Peprotech) and three days later, medium was again changed to DMEM supplemented with M-CSF (25 ng/mL), sRANKL (50 ng/mL; Peprotech), dexamethasone (10 nM; Sigma Aldrich) and TGF-β (2.5 ng/mL; R&D Systems) in order to differentiate the osteoclast precursors into mature osteoclasts. The culture medium was then changed twice a week. Adherent cells at day 1 and cells cultured on bone slices for 7, 14 and 21 days [13] were used for functional assays and gene expression.

### Functional assays

Tartrate-resistant acid phosphatase (TRAP) staining of OCs was performed at days 7, 14 and 21 of culture using the Acid Phosphate Leukocyte Kit (TRAP, Sigma-Aldrich) according to the manufacturer's instructions. OCs were counted as TRAP positive cells containing three or more nuclei [14,15]. For measurement of resorbed area in the resorption assay at days 7, 14 and 21 of culture, cells were removed from the bone slices using sodium hypochlorite and stained with 0.1% toluidine blue [16]. Bone slices from both TRAP staining and resorption functional assays were photographed in an area of 1.25 mm^2^ with a brightfield microscope (Leica DM2500, Leica) under a 10x objective. The number of TRAP stained OCs was counted for each time-point per condition and the resorption pits were traced using ImageJ software (NIH, Bethesda, MD). The resorbed area was calculated and expressed in % of total area.

### Gene expression

RNA was extracted from cells cultured over the bone slices at days 1, 7, 14 and 21 of culture using NZYol (NZYTech) according to the manufacturer's instructions. Following RNA extraction, total RNA concentration and purity was quantified using Nanodrop 1000 (Thermo Scientific). Complementary (c)DNA was synthesized at a concentration of 0.6 ng/μL using the DyNAmo^™^ cDNA Synthesis Kit (Thermo Scientific) according to the manufacturer's instructions. Genes that encode for osteoclast proteins such as CSF1R, RANK, NFATc1, ATP6V0D2 and CTSK were studied (see Table 1 for primers) by real-time quantitative PCR (RT-qPCR). Ribossomal RNA 18s was chosen as the housekeeping gene. Primers were designed using the primer-BLAST [17] software and qPCR was performed using the DyNAmo^™^ Flash SYBR Green qPCR Kit (Thermo Scientific). The efficiency of qPCR was analysed using the standard curve method [18] as described previously [19]. The values obtained were normalized with the housekeeping gene 18s rRNA.

**Table 1:** Primers used for osteoclast gene expression.

Gene	Primer sequence	Transcript size
CFSR1	Fw 5'—GAACATCCACCTCGAGAAGAAA—3'	88bp
	Rev 5'—GACAGGCCTCATCTCCACAT—3'	
RANK	Fw 5'—GAACATCATGGGACAGAGAAATC—3'	89bp
	Rev 5'—GGCAAGTAAACATGGGGTTC—3'	
NFATc1	Fw 5'—GCAAGCCGAATTCTCTGGTG—3'	144bp
	Rev 5'—TACCGTTGGCGGGAAGGTAG—3'	
ATP6V2D0	Fw 5'—CATTCTTGAGTTTGAGGCCG—3'	186bp
	Rev 5'—CCGTAATGATCCGCTACGTT—3'	
CTSK	Fw 5'—GCCAGACAACAGATTTCCATC—3'	75bp
	Rev 5'—CAGAGCAAAGCTCACCACAG—3'	
18s rRNA	Fw 5'—GGAGTATGGTTGCAAAGCTGA—3'	129bp
	Rev 5'—ATCTGTCAATCCTGTCCGTGT—3'	

Annealing temperature for all primers was 60°C

### Statistical analysis

Statistical analysis was performed with SPSS Statistics 17.0 (IBM). Categorical variables were expressed as frequencies and comparisons were tested using chi-square test. Continuous variables were expressed by median and interquartile range. Baseline and post-treatment (follow-up) values within each sample were compared using Wilcoxon's matched-pairs signed-rank test. To compare AS patients with healthy age and sex-matched donors Mann-Whitney test was used. Correlation analysis was performed using Spearman's correlation coefficients. Values were corrected for multiple comparisons and p-values lower than 0.05 were considered significant.

## Results

### Patient background

Thirty-eight subjects were recruited, including 13 AS patients, evaluated before and after TNFi therapy, and 25 age and gender matched healthy donors. Despite having an initial cohort of 25 patients 3 were lost for follow-up and 9 switched biological therapy at 3 months follow-up. Patients were treated with one of the four TNFi currently used in clinical practice: Adalimumab (n = 1, 8%), Golimumab (n = 5, 38.5%), Infliximab (n = 2, 15%) or Etanercept (n = 5, 38.5%). Treatment duration ranged from a minimum of 6 up to 12 months. The clinical and demographic characteristics of the patients both at baseline and follow-up and healthy donors are described in Table 2.

**Table 2:** Summary of the patients and healthy controls' characteristics.

	AS patients		Healthy	p-value
	Baseline	Follow-up		
Sample size	13		25	
Age (years)	37 [33–43]		39 [36–49]	0.8028
Females %	38%		48%	0.7342
Symptoms duration (years)	10 [7–21]		NA	
HLA-B27 (% positive)	54%		NA	
Presence of syndesmophytes (%)	40%		NA	
Peripheral involvement (%)	46%		NA	
Treatment with NSAIDs (%)	77%		NA	
NSAIDs duration (months)	24 [8–42]		NA	
Treatment with DMARDs (%)	46%		NA	
DMARDs duration (months)	24 [7–47]		NA	
Treatment with corticosteroids (%)	15%		NA	
ESR (mm/h)	30 [14–54]	7 [4–17]	NA	0.0010*
CRP (mg/dl)	1.4 [0.1–3.0]	0.1 [0.0–0.6]	NA	0.0034*
ASDAS	3.8 [2.2–4.3]	1.7 [1.4–1.9]	NA	0.0001*
BASDAI	4.7 [3.9–7.5]	2.5 [1.5–4.1]	NA	0.0007*
BASFI	6.2 [5.1–7.4]	3.9 [1.2–5.4]	NA	0.0032*
TNFi duration (months)	NA	12 [6–12]	NA	

Data is represented as median [Interquartile range] unless stated otherwise; DMARDs include methotrexate, hydroxychloroquine and sulfasalazine; AS—ankylosing spondylitis; HLA—human leukocyte antigen; NA—not applicable; NSAIDs—non-steroidal anti-inflammatory drugs; DMARDs—disease-modifying antirheumatic drugs; ESR—erythrocyte sedimentation rate; CRP—C-reactive protein; ASDAS—ankylosing spondylitis disease activity score; BASDAI—Bath ankylosing spondylitis disease activity index; BASFI—Bath ankylosing spondylitis functional index; TNFi—tumor necrosis factor inhibitors.

* p-value<0.05.

### TNFi treatment in AS patients decreases the number of RANKL^+^ T and B cells in circulation

**Fig 1:**
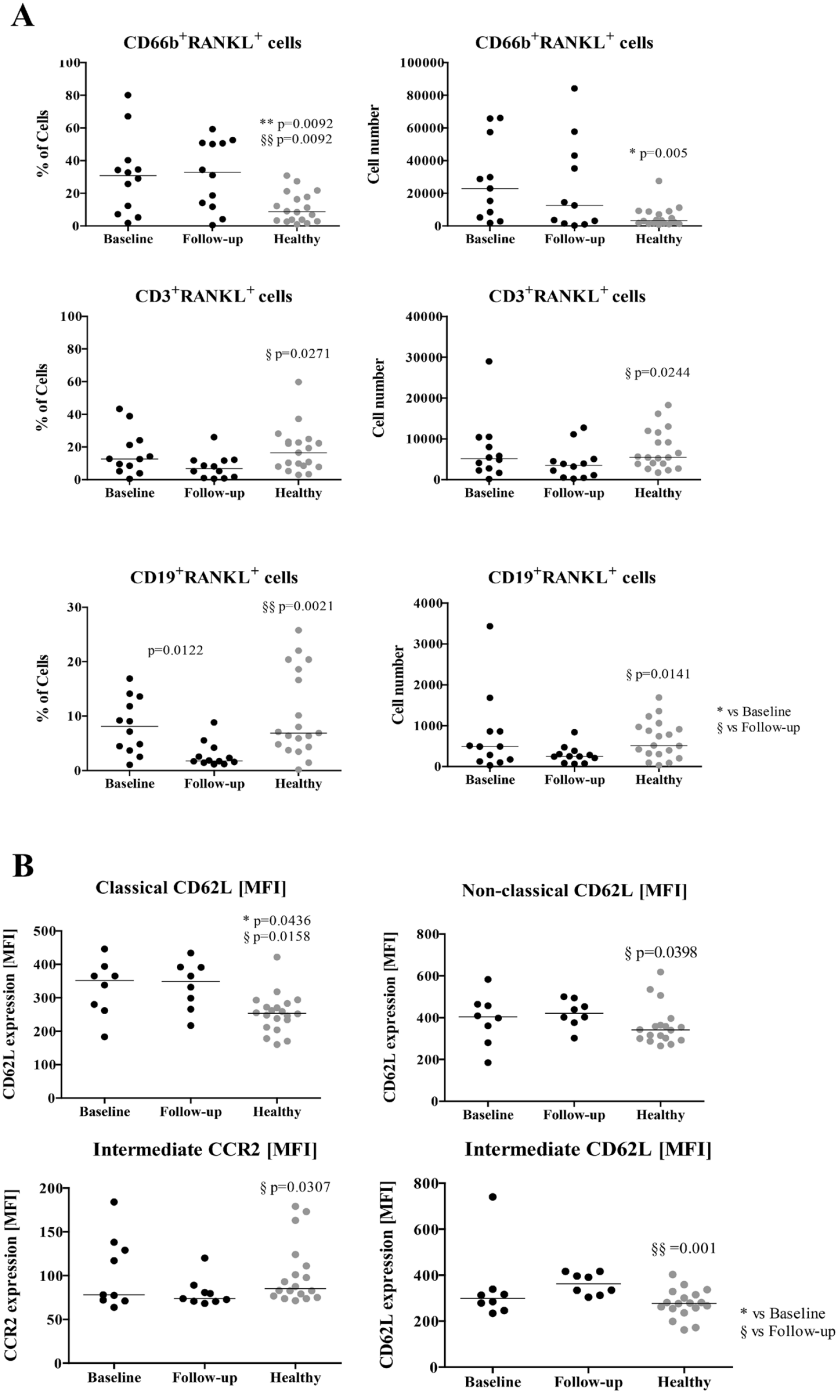
RANKL surface expression in leukocytes and monocyte phenotype. A) RANKL^+^ frequency and absolute number in circulating leukocytes. CD66b^+^RANKL^+^ cells are increased in the circulation of patients at baseline and at follow-up when compared to healthy donors (frequency p = 0.0092; absolute number p = 0.005). At follow-up, CD3^+^RANKL^+^ are decreased in circulation when compared to healthy donors (frequency p = 0.0271; absolute number p = 0.0244). CD19^+^RANKL^+^ frequency is decreased after treatment when compared to patients at baseline (p = 0.0122) and with healthy donors (p = 0.0021). This difference is also observed at the absolute number level when compared to healthy donors (p = 0.0141). RANKL^+^ cells were analysed by flow cytometry and gated inside each subpopulation. B) Phenotype of circulating monocyte subpopulations—Classical CD14^bright^CD16^-^, intermediate CD14^bright^CD16^+^, non-classical CD14^dim^CD16^+^. CD62L is increased in the circulating classical subpopulation of patients at baseline (p = 0.0436) and at 6 months follow-up (p = 0.0158) when compared to healthy donors. CD62L expression is increased in patients after 6 months follow-up both in the non-classical subpopulation (compared to healthy p = 0.0398) and in the intermediate subpopulation (compared to healthy p = 0.001). CCR2 expression is reduced in the intermediate subpopulation of patients at follow-up when compared to healthy donors (p = 0.0307). Each dot represents a sample. Line represents median. * vs Baseline, § vs Follow-up. * and § p<0.05, ** and §§ p<0.01. MFI—Median fluorescence intensity.

RANKL surface staining was performed in whole blood leukocytes (neutrophils—CD66b^+^; T cells CD3^+^; B cells CD19^+^; Fig 1A). No difference was found in the total number of circulating neutrophils, T or B cells before or after therapy or when compared to healthy donors (data not shown). However, CD66b^+^RANKL^+^ cells were higher in patients than in healthy donors, both at baseline and follow-up. After TNFi therapy patients had lower number of CD3^+^RANKL^+^ cells in circulation when compared to healthy donors, both in percentage and absolute number (p = 0.0271 and p = 0.0244, respectively; Fig 1A). Furthermore CD19^+^RANKL^+^ cell frequency and absolute number was decreased in patients after TNFi treatment (percentage value significantly different, p = 0.0122; Fig 1A).

Regarding monocyte subpopulations (classical CD14^bright^CD16^-^, intermediate CD14^bright^CD16^+^ and non-classical CD14^dim^CD16^+^) no differences in frequency or cell death among the three subpopulations and between groups were found. CD62L, a cell adhesion molecule also known as L-selectin, was increased in the circulating classical subpopulation of patients, both at baseline and follow-up, when compared to healthy donors (p = 0.0436; p = 0.0158; Fig 1B). Moreover, comparing with healthy donors, CD62L expression was higher in patients after 6 months of TNFi therapy both in the non-classical subpopulation (p = 0.0398; Fig 1B) and in the intermediate (p = 0.001; Fig 1B) subpopulations. Conversely, CCR2 expression was lower in the intermediate subpopulation in patients after TNFi when compared to healthy donors (p = 0.0307; Fig 1B). No differences were identified in any of the other studied surface markers.

### IL-17A, IL-23 and TGF- β circulating levels are reduced in AS patients after TNFi treatment

**Fig 2:**
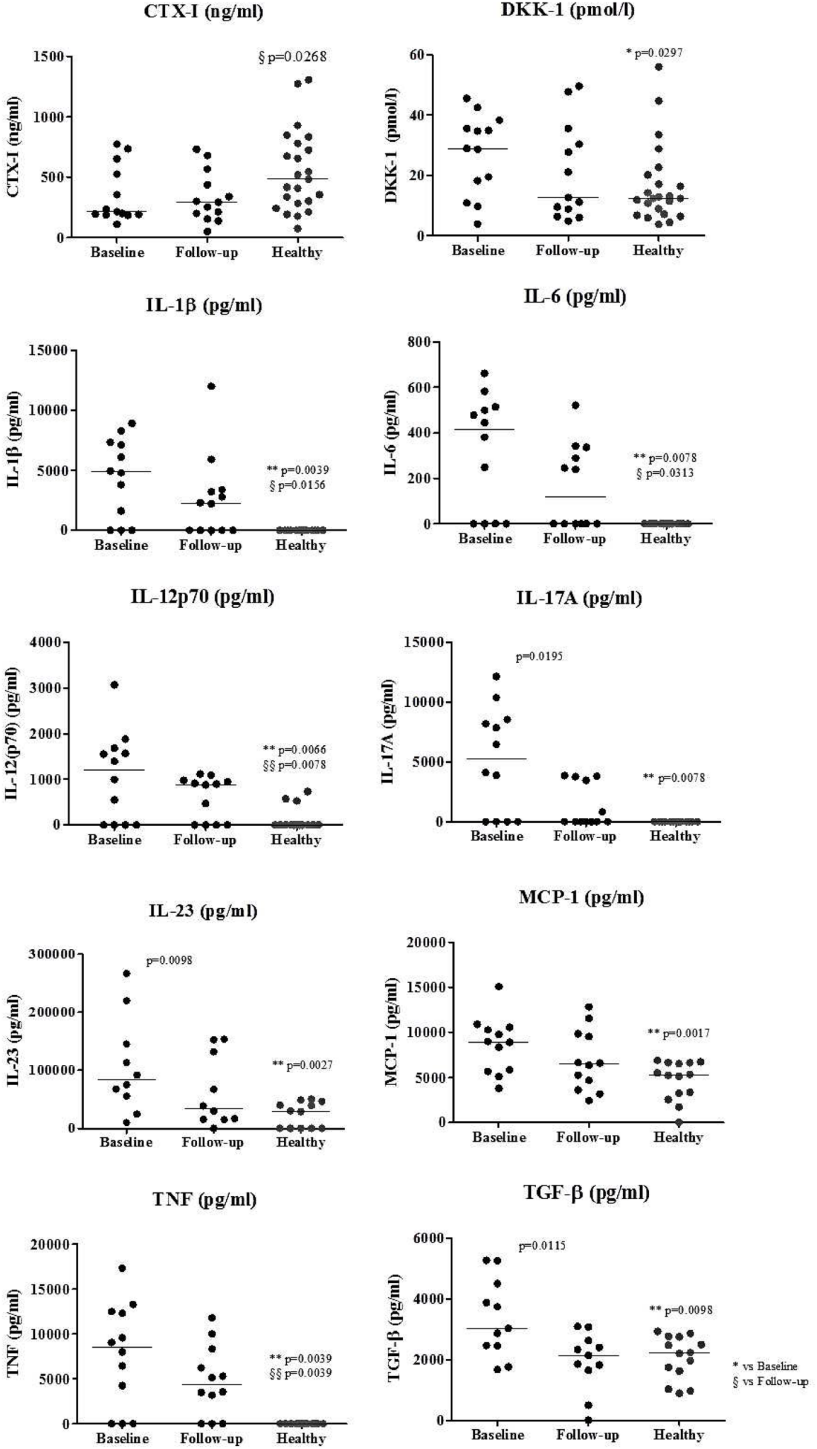
Serum levels of bone turnover markers, bone metabolism proteins and cytokines. CTX-I levels are decreased in patients at 6 months follow-up when compared to healthy donors (p = 0.0268). DKK-1, IL-1 β, IL-6, IL-17A, IL-12p70, IL-23, TNF, MCP-1 and TGF-β are increased in patients at baseline when compared to healthy donors. After 6 months of therapy, follow-up patients had decreased levels of IL-17A, IL-23 and TGF-β when compared to their baseline. We have also observed that after therapy the levels of IL-1 β, IL-6, IL-12p70 and TNF were still significantly higher than healthy donors levels’. Each dot represents a sample. Line represents median. * vs Baseline, § vs Follow-up. DKK—dickkopf-related protein, CTX—carboxy-terminal collagen crosslinks, IL—interleukin, TNF—tumor necrosis factor, MCP—monocyte chemmotractant protein, TGF—transforming growth factor.

DKK-1, IL-1β, IL-6, IL-17A, IL-12p70, IL-23, TNF, MCP-1 and TGF-β levels were significantly higher in patients at baseline when compared to healthy donors (Fig 2). After correcting for multiple comparisons only IL-1β, IL-23, MCP-1 and TNF remained significantly higher in patients at baseline when compared to healthy donors.

Circulating levels of IL-17A, IL-23 and TGF-β were decreased after TNFi treatment when compared to baseline (p = 0.0195, p = 0.0098 and p = 0.0115, respectively; Fig 2). CTX-I levels were lower in patients at 6 months of follow-up when compared to healthy donors (p = 0.0268; Fig 2). After correcting for multiple comparisons, none of the markers were significantly decreased after treatment.

### Osteoclast differentiation from circulating precursors in AS patients is lower than in healthy controls and osteoclast-mediated bone resorption is increased after TNFi treatment

**Fig 3:**
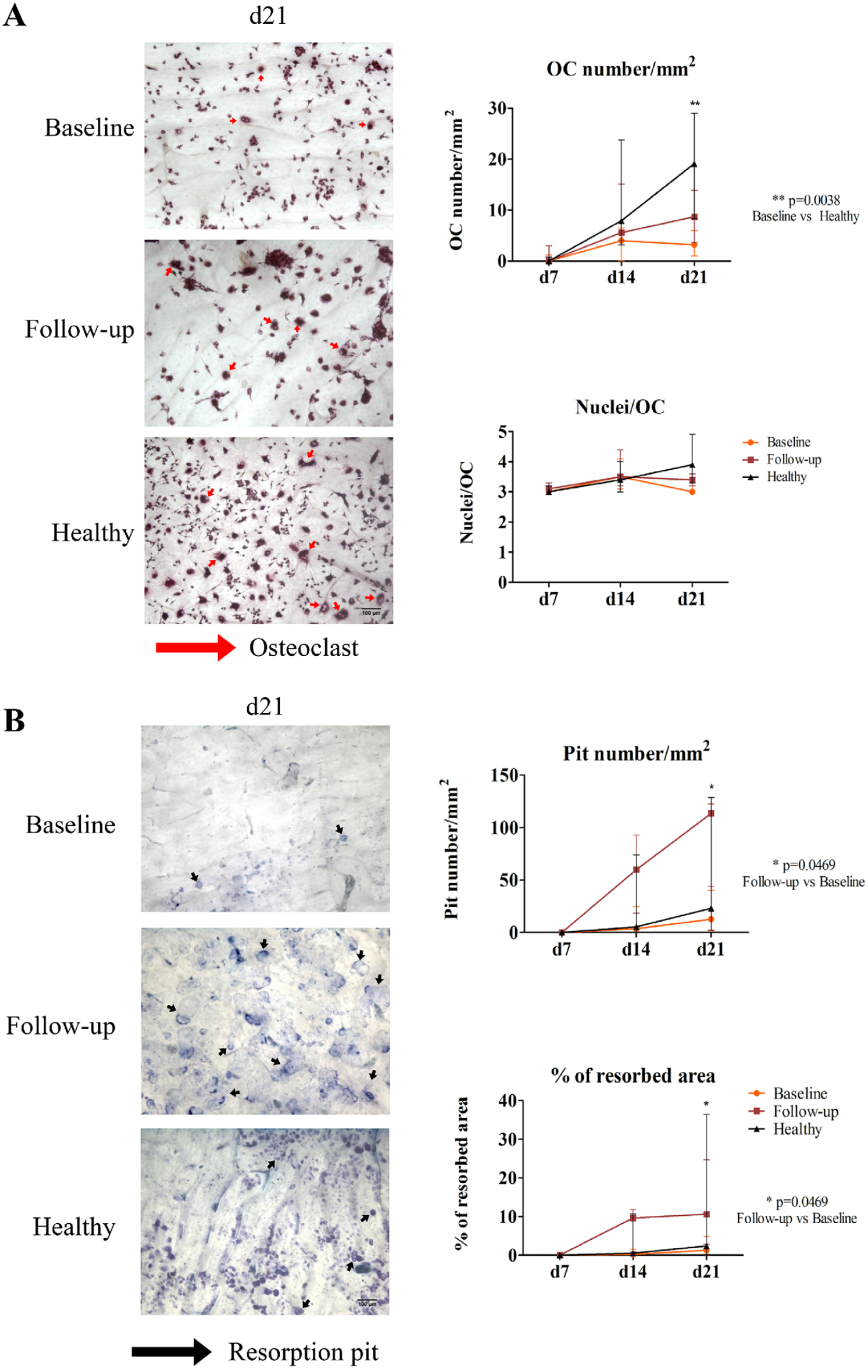
Osteoclast number is reduced in baseline patients, but bone resorption activity is increased after TNF-blocker exposure. A) Representative images of culture day 21 of cells stimulated with M-CSF, RANKL, dexamethasone and TGF-β and stained for TRAP. OC number increased throughout time and at culture day 21 baseline patients have significantly less osteoclasts than healthy donors (p = 0.0038). No differences were found in the number of nuclei per OC in any studied time of culture. B) Representative images of pit assay at culture day 21. Patients at follow-up had significantly higher number of pits and resorption area at culture day 21 when compared to their baseline (p = 0.0469 for both resorption pit number and percentage of resorbed area). Dots represent median counts for each group at each time-point and bars represent interquartile range [10–90]. d—day; OC—osteoclast; Scale bars 100μm, red arrows—osteoclasts, black arrows—resorption pits.

Under stimulation, reproducing local bone inflammatory microenvironment, AS patients, prior to TNFi, had less OC differentiation at culture day 21 than healthy donors (p = 0.0038; Fig 3A). However, the number of OC increased throughout the culture period in all the studied groups. No differences were found in the number of nuclei per osteoclast between the studied groups. Both resorption pit number and percentage of resorption area were markedly increased after culture day 14 in cells from patients treated with TNFi as compared to baseline, reaching statistical significance at culture day 21 (p = 0.0469 for both resorption pit number and percentage of resorbed area; Fig 3B).

**Fig 4:**
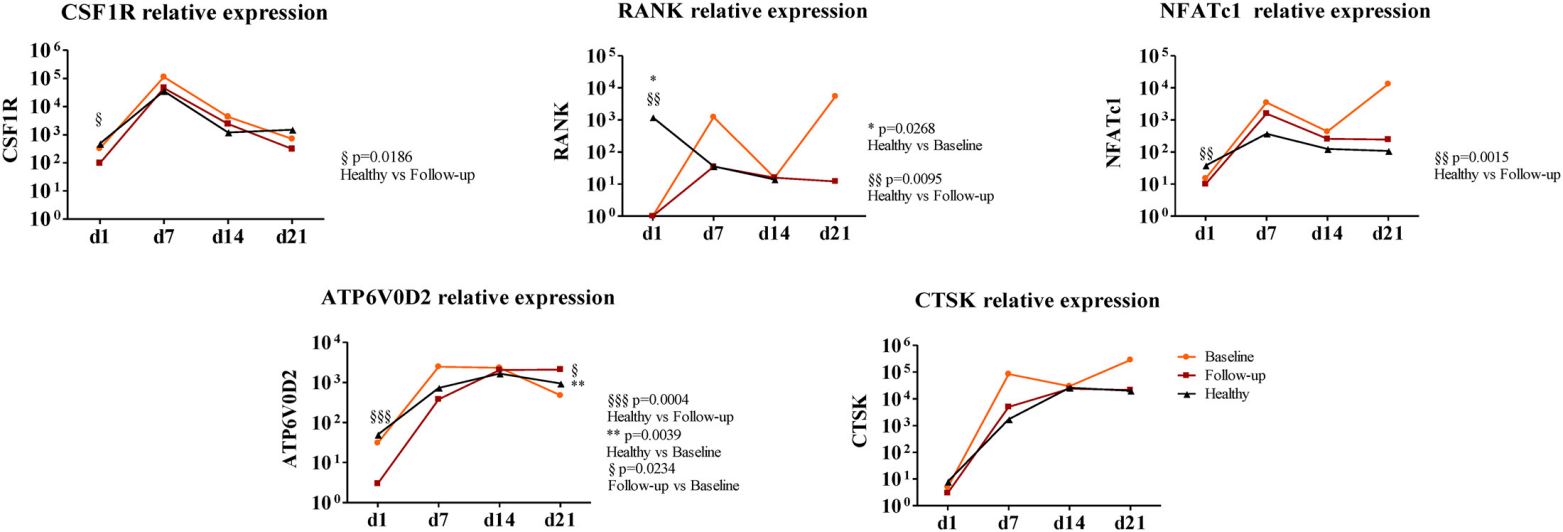
Gene expression profile of stimulated cells in culture for 21 days. All genes except CTSK are significantly decreased at day 1 in patients at follow-up when compared to healthy donors. At day 1 RANK expression in patients at baseline is also significantly reduced when compared to healthy donors (p = 0.0268). We found no differences between groups throughout the culture time except for Atp6v0d2 expression at day 21 in patients at baseline which is significantly reduced when compared both to healthy donors (p = 0.039) and patients at follow-up (p = 0.0234). Relative gene expression shown in Log scale. Dots in graphs represent median gene expression for each group at each time-point. d—day; CSF1R—gene encoding macrophage-colony stimulating factor (c-fms), RANK—gene encoding for receptor activator of nuclear factor-κβ, NFATc1—gene encoding for nuclear factor of activated T-cells, Atp6v0d2—gene encoding ATPase, H^+^ transporting, lysosomal V0 subunit D2, CTSK—gene encoding cathepsin K. p<0.05 is considered significant.

Gene expression by RT-qPCR was performed for OC genes that are known to be important during its differentiation and activity. All genes, except CTSK, were significantly lower at culture day 1 in patients after TNFi treatment when compared to healthy donors (CSF1R p = 0.0186; RANK p = 0.0095; NFATc1 p = 0.0015; ATP6V0D2 p = 0.0004; Fig 4). At culture day 1 RANK expression in patients at baseline was significantly lower than in healthy donors (p = 0.0268; Fig 4). There were no differences between patients and controls or between baseline and post TNFi treatment patients, except for ATP6V0D2 expression at culture day 21 in patients at baseline, which was significantly lower than in healthy donors (p = 0.039; Fig 4) and than in patients after TNFi (p = 0.0234). After correcting for multiple comparisons, expression of RANK, NFATc1 and ATP6V0D2, in culture day 1, from patients after TNFi remained significantly lower than in healthy donors.

No differences were found in any of the studied parameters when comparing presence or absence of HLA-B27, presence or absence of peripheral involvement or comparing monoclonal antibodies (Adalimumab, Infliximab or Golimumab) to the fusion protein Etanercept (data not shown).

## Discussion

We have shown that in TNFi treated AS patients, the pro-inflammatory and pro-osteoclastogenic systemic stimuli were decreased due to reduced RANKL^+^ circulating lymphocytes (B and T cells) and reduced levels of IL-17A and IL-23. Accordingly, OC specific gene expression was reduced in circulating precursors after TNFi exposure. However, when these precursors from TNFi treated AS patients were cultured in OC differentiating conditions, reproducing the bone microenvironment, their response to osteoclastogenic stimuli and activity was increased in comparison to baseline behavior.

One of the limitations of our study is the sample size and these results should be confirmed in a larger population. Another limitation is the use of circulating precursors. Bone local samples would have been preferred to use, however surgery to this bone areas of interest are rare and in addition it would be most difficult to obtain healthy controls for these samples. Our strategy was to address the question of circulating precursors being an important source of osteoclasts in active disease and the fate of their osteoclast differentiation ability after exposure to TNFi.

Our study found no correlation between monocyte phenotype, osteoclast activity or gene expression and treatment duration. Although there have been suggestions that longer therapy duration increases bone mineral density in AS patients [20] these studies have been performed in longer time courses than our study. We also found no differences between the use of monoclonal antibodies (Adalimumab, Golimumab and Infliximab) and the recombinant protein Etanercept. Most of the literature does not compare different TNFi [21,22] and thus more studies are needed to address if differences might exist at the level of radiographic progression.

Recent studies and meta-analysis have shown that TNFi treatment in AS patients is associated with increased lumbar and hip BMD [23]. In addition, an increased likelihood of developing new bone following resolution of inflammation after TNFi therapy has been suggested. Accordingly, radiographic progression was associated with decreased systemic inflammation and, on the contrary, radiographic nonprogression was associated with persistent inflammation, as assessed by IL-6 and CRP levels and MRI, supporting a link between the resolution of inflammation and new bone formation in AS patients during TNFi therapy [24,25].

It has been previously reported that neutrophils are more active in AS patients [26] but no differences in total B or T cell numbers were reported [27]. In our study no differences were found in the frequency of granulocytes and T and B cells between any of the studied groups. However, RANKL^+^ neutrophils count was increased in patients at baseline and TNFi treatment reduced the number of RANKL^+^ T and B cells. Previous studies have shown that T lymphocytes from AS patients have higher expression of RANKL than healthy donors [28], but to our knowledge a comparative study of RANKL expression in AS patients before and after TNFi was never been published.

In previous studies AS patients under NSAID therapy have been showed to have increased circulating number of classical monocytes and decreased non-classical monocytes when compared to healthy donors [29]. However, in our cohort, no differences were detected in any of the circulating monocytes subpopulations. In the intermediate monocyte subpopulation, patients exposed to TNFi had decreased CCR2 expression. CCR2 is a chemokine receptor that binds MCP-1 and promotes osteoclast precursors fusion and maturation [30]; its reduction after treatment is in accordance with the reduced gene expression of specific osteoclast genes observed in cells from patients after TNFi treatment and with its previously described role in osteoclastogenesis [30,31]. On the other hand, CD62L (L-selectin) was higher in AS patients after TNFi therapy in all three monocyte subpopulations when compared to healthy donors suggesting that adhesion is increased in these cells after exposure to TNFi. We speculate that our observation of high L-selectin (CD62L) expression in circulating monocytes subpopulations after TNFi treatment may be related to increased adhesion of OC precursors to bone slices and subsequent cell activation. It was previously described in rats that the binding of L-selectin to some of its ligands (namely GlyCAM-1) increases integrin binding (β2 and also α4) [32–34]. Therefore, binding of L-selectin to ligands on the bone slice might increase αvβ3 integrin binding leading to increased OC differentiation [35]. We suggest that when osteoclast precursors attach to bone slices through integrins and L-selectin (CD62L), signaling pathways are activated and the expression of OC differentiation genes is induced. It has been previously shown that attenuation of the integrin α_V_β_3_ pathway leads to inhibition of OC differentiation and that there is a crosstalk between integrin β_3_ and M-CSF/c-fms pathways [36]. We further suggest that cell adhesion to bone by integrins plays an important role in OC differentiation, but additional studies are required to determinate how integrins are able, *per se*, of inducing OC differentiation.

As previously reported, serum levels of IL-1β, IL-6, IL-12p70, IL-17A, IL-23, MCP-1, TGF-β and TNF were significantly higher in AS patients with active disease when compared to healthy subjects [37,38]. In addition, IL-17A, IL-23 and TGF-β were significantly decreased in AS patients after TNFi therapy. IL-17A and IL-23 are well known for their role in the pathogenesis of inflammatory disorders, such as AS [39], and TGF-β is an important cytokine for bone formation [40]. In accordance with our study, Limón-Camacho *et al* found that serum levels of IL-17A were significantly elevated in AS patients with active disease, when compared to patients receiving TNFi; the same findings were observed for IL-6, IL-12, and TNF [37]. Moreover, we observed that the levels of most cytokines present in the serum of AS patients normalized to the healthy donors levels after 6 months of TNFi therapy, indicating a reduction in the inflammatory environment induced by TNF blockade. Concordantly, patients under TNFi had reduced levels of CTX-I, suggesting a decrease in osteoclast activity. However, no differences in the levels of sRANKL, OPG and their ratio, before and after TNFi were found. Previous studies have shown discrepancies in sRANKL and OPG levels in AS patients. While sRANKL and OPG have been found increased in AS patients with active disease [41], in patients with mild to active disease sRANKL/OPG was lower than in healthy controls [38,42]. These latter studies found that after TNFi, sRANKL/OPG ratio was increased due to a decrease in OPG; however, none of the studies used paired patients samples.

SOST and DKK-1 have been implicated in the pathophysiology of AS, either by their reduced expression or by their functional de-regulation [43]. In our study, DKK-1 serum levels were higher in patients at baseline when compared to healthy controls; however, no significant differences in SOST or DKK-1 serum levels after treatment were found. Taylan *et al* reported that patients under TNFi presented higher DKK-1 levels compared to patients under conventional therapy (NSAIDs and/or DMARDs) [38]. More recently, Ustun *et al* found that TNFi did not affect DKK-1 and SOST levels [44]. However, several other studies suggested that DKK-1 levels decrease after TNFi and that there is no change in SOST circulating levels [43].

To understand the effect of TNFi in osteoclast differentiation and function we isolated PBMCs from AS patients before and after TNFi and cultured them *in vitro* over bone slices. OC formation continuously increased up to day 21. Both controls and patients (before and after TNFi treatment) exhibited the same pattern of increase in the number of resorption pits and the percentage of resorbed area over time, although at day 14 there was a marked increase in resorption in the TNFi treated patients that reached statistical significance at day 21. After TNFi treatment no differences in pit size were found suggesting that OC mobility is not affected by the disease or therapy and we found no differences in the number of nuclei/osteoclast, which has been suggested to correlate with osteoclast activity [45–47].

There is evidence that TNF contributes to expression of specific OC proteins and that it directly activates OC differentiation through cross activation of the NF-κB pathway or c-Jun N-terminal kinase (JNK) signaling cascade [48]. In our study, all genes, with the exception of cathepsin K, were downregulated after TNFi treatment. However, this reduced gene expression did not impair OC differentiation when the PBMCs were cultured under OC differentiating conditions. At later time points, culture days 14 and 21, there was a significant increase in ATP6V0D2 expression in healthy and TNFi treated patients, which might be related to our observation of increased bone resorption after TNFi.

In summary, in AS patients TNFi treatment reduces systemic pro osteoclastogenic stimuli. However, TNFi effect on OC precursors from AS patients increases their response to osteoclastogenic stimuli and their activity in bone pro inflammatory microenvironment. This is in disagreement with the apparent increase in osteoproliferation in AS patients treated with TNFi [24,25]. However, patients treated early in the course of the disease appear to escape this effect [49]. We hypothesize that early TNFi treated patients have an early normalization of bone resorption by TNFi, thus preventing osteoproliferation.
